# Multi-focus microscope with HiLo algorithm for fast 3-D fluorescent imaging

**DOI:** 10.1371/journal.pone.0222729

**Published:** 2019-09-20

**Authors:** Wei Lin, Dongping Wang, Yunlong Meng, Shih-Chi Chen

**Affiliations:** 1 Department of Mechanical and Automation Engineering, The Chinese University of Hong Kong, Shatin, Hong Kong; 2 Institute of Modern Optics, Tianjin Key Laboratory of Optoelectronic Sensor and Sensing Network Technology, Nankai University, Tianjin, China; Texas A&M University, UNITED STATES

## Abstract

In this paper, we present a new multi-focus microscope (MFM) system based on a phase mask and HiLo algorithm, achieving high-speed (20 volumes per second), high-resolution, low-noise 3-D fluorescent imaging. During imaging, the emissions from the specimen at nine different depths are simultaneously modulated and focused to different regions on a single CCD chip, i.e., the CCD chip is subdivided into nine regions to record images from the different selected depths. Next, HiLo algorithm is applied to remove the background noises and to form clean 3-D images. To visualize larger volumes, the nine layers are scanned axially, realizing fast 3-D imaging. In the imaging experiments, a mouse kidney sample of ~ 60 × 60 × 16 μm^3^ is visualized with only 10 raw images, demonstrating substantially enhanced resolution and contrast as well as suppressed background noises. The new method will find important applications in 3-D fluorescent imaging, e.g., recording fast dynamic events at multiple depths in vivo.

## Introduction

Fast 3-D fluorescent imaging has been one of the most important research areas in modern biophotonics. It is realized by 3-D data acquisition and image reconstruction. Several approaches have been proposed for volumetric imaging, including point scanning 3-D microscopy [1–3], holographic 3-D microscopy [4, 5] and wide field microscopy [6–8] etc. Although point scanning systems provides the highest resolution, the data acquisition process is sequential and time-consuming, making large volume fast imaging impractical. Holographic 3-D microscopy has the advantage of fast data acquisition, i.e., a volume image can be reconstructed based on three wide field holograms. However, the high imaging speed is realized at the expense of low axial resolution [9]. Wide field microscopy strikes a good balance between speed and resolution, and thus has been extensively used and investigated. A wide field system sequentially collects 2-D images along the optical axial and assembles the collected images to form volumetric images [10]. The temporal resolution of wide field microscopy is generally limited by the sequential axial scanning process.

Recently, several microscopy methods have been proposed to address this issue [11–15]. Among them, multi-focus microscopy (MFMs) is an approach that can simultaneously image multiple focal planes at non-overlapping adjacent sections of a camera chip via a single exposure; as such, dynamic events at different focal planes in a volume can be recorded simultaneously. Conventionally, multi-focal plane imaging can be realized via a few different means, e.g., introducing a diffractive optical element in the Fourier plane of the optical system to function as a multifocal grating (MFG) [11, 12], volume holographic grating (VHG) [13], multi-plane prism [14] or quadratically distorted grating (QDG) [15]. Comparing with the static components, a programmable spatial light modulator (SLM) presents a more versatile and capable solution for MFM. For example, an SLM can arbitrarily and instantaneously adjust the multiplexed number and spacing by using a phase pattern superposing with multiple off-axis Fresnel lenses of different focal lengths [16].

Similar to other wide field systems, the performance of MFM is often plagued by the background noises, i.e., excited out-of-focus fluorescent signals, which deteriorates the image quality. Several methods have been introduced to address this issue, including the 3-D deconvolution and structured light illumination [17, 18]. One disadvantage of the deconvolution method is that it requires the characterization of the system’s 3-D point spread function (PSF) and the measurement needs to be repeated when the number and spacing of the focal planes are changed, which calls for a flexible device such as SLM. In comparison, structured illumination algorithms [18–21] can be directly applied to MFM to enhance the imaging results without prior knowledge of PSF. However, the method compromises the temporal resolution as several raw images are required to reconstruct one image. And the more raw images the algorithm requires, the lower imaging speed becomes. Hence, the HiLo algorithm [22–24], which only involves two raw images for image reconstruction, is a good candidate for realizing background rejection in MFM.

In this paper, we present a new MFM for fast volumetric imaging based on the HiLo algorithm, where programmable multi-focal imaging is achieved via an SLM. The pattern on the SLM is designed by superposing a phase pattern with off-axis Fresnel lenses of different focal positions and optimized by the modified Gerchberg-Saxton algorithm, thereby allowing the generation and control of multiple focal planes in space. HiLo algorithm is introduced to enhance the image contrast at each focal plane and suppress background noises. The sectioning performance of the HiLo-MFM system with varying focal plane spacing and position has been studied. In the experiment, high-contrast 3-D images of a mouse kidney is successfully reconstructed, verifying the speed and resolution of the HiLo-MFM system. The new HiLo-MFM may find important applications in fast 3-D fluorescent imaging, e.g., visualizing dynamic biological events at multiple depths in vivo.

### Experimental setup and working principle

**Fig 1:**
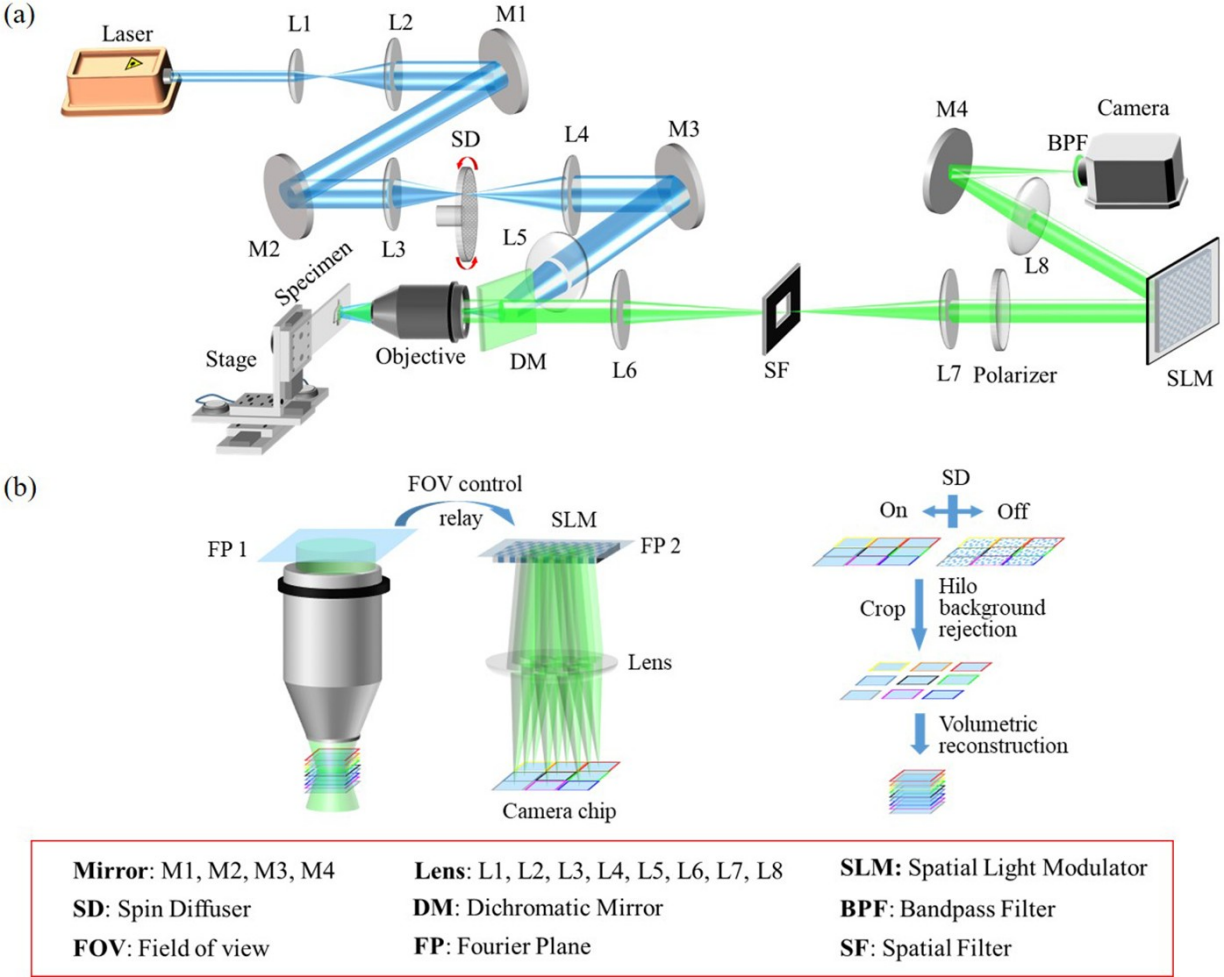
Experimental setup and working principle of HiLo-MFM (a) Optical configuration of the HiLo-MFM system; M1 –M4, mirrors; L1 –L8, lenses; SLM, spatial light modulator; SD, spin diffuser; DM, dichroic mirror; BPF, band pass filer; FOV, field of view; FP, Fourier plane; SF, spatial filer; (b) working principle of HiLo-MFM.

Fig 1(A) presents the optical configuration of the HiLo-MFM system. The light source is a 488 nm continuous-wave laser (MLD 488 nm, Cobolt Inc.). For illumination, the laser beam is first expanded and focused on a diffuser (SUSS MicroOptics, diffuser Nr. 13–00027) via L3 (*f* = 150 mm), where the laser is modulated by random phase distribution and relayed to the back aperture of the objective lens (CFI Apo LWD 40X; NA 1.15, Nikon). The laser can be switched between speckle and uniform illumination by controlling the diffusor. The emission is collected by the same objective and separated from the reflected illumination light via a dichroic mirror (DMLP490R, Thorlabs). The sample is mounted on an xyz stage (MP285, Sutter Instrument) for positioning. Through a tube lens L6 (*f* = 150 mm), the sample is imaged onto its conjugation plane where a spatial filter sets the field of view. Next, a 4-*f* system relays the Fourier plane of the objective to a liquid crystal-based spatial light modulator with a reflectivity of 67% and diffraction efficiency of ~80% (LC-SLM, Pluto-2-VIS-016, Holoeye). Because the back aperture diameter of the objective is 11.43 mm which is larger than the width of SLM chip, i.e., 8.64 mm, the focal length of tube lens L7 is selected to be 100 mm such that all frequencies in the Fourier plane of the objective can be modulated by the SLM. A polarizer is placed before the SLM to modulate the emission into linearly polarized light. Lastly, after passing through a condenser lens L8 (*f* = 200 mm), the emission is imaged on the camera chip, i.e., Scientific Complementary Metal-Oxide-Semiconductor (sCOMS, ORCA-Flash4.0 LT C11440-42U30, Hamamatsu). The overall magnification of the imaging system is 60. A bandpass filter (BPF, 10BPF25-550, Newport. Inc.) is used to select the target emission signals and reduce the chromatic aberration introduced by the SLM.

Fig 1(B) illustrates the working principle of the HiLo-MFM. The specimen is wide-field illuminated, and the fluorescence of a large volume is excited and collected by the high NA objective. The Fourier plane of the objective is then relayed to the SLM after field of view control. The SLM modulates the fluorescence by displaying a phase mask that superposes multiple off-axial Fresnel lenses phase with different focal lengths. After the SLM modulation, different focal depths images focus on different parts of the camera chip. In this regard, the depth of field multiplexing is achieved. When the spin diffusor is rotating, a raw image under uniform illumination is obtained; while spin diffusor is static, a raw image under speckle illumination is obtained. These two raw images are cropped and used to reconstruct image sections with background rejection. As can be seen, the calculation of SLM phase pattern for multi-focus multiplexing and the HiLo background rejection algorithm are the two most significant techniques in fast volumetric image based on HiLo-MFM and will be discussed in detail in the following two sections.

### Calculation of phase patterns

The phase mask pattern is designed by superposing a phase pattern with off-axis Fresnel lenses of different focal positions and optimized by the iterative Fourier transform algorithm, i.e., the weighted global Gerchberg-Saxton (GS) algorithm, which is derived from the weighted GS and global GS algorithms [25–27]. The algorithm is summarized in Table 1, where F(E) and $F^{‐1}$ (*E*) denote the Fourier transform and inverse Fourier transform of an optical field *E* respectively, and T(E,ΔL) is the transform process of an optical field *E* propagating over a distance of *ΔL*. F(E) and T(E,ΔL) can be mathematically expressed as follows:

$$U(u,v)=F(E(x,y))=\frac{1}{i\lambda f}\iint E(x,y)\exp(\frac{i2\pi (ux+vy)}{\lambda f})dxdy$$ (1)

$$E_{z}(x,y)=T\left(E(x',y'),\Delta L\right)=\iint \frac{E(x',y')}{\sqrt{i\lambda \Delta L}}\exp\left[\frac{i\pi ({\left(x-x'\right)}^{2}+{\left(y-y'\right)}^{2})}{\lambda \Delta L}\right]dx'dy'$$ (2)

where *λ* is the wavelength; *f* is the focal length; Δ*L* is the propagation distance; In this work, the central wavelength of the emission signals is determined by the bandpass filter (550 nm); as L8 is the Fourier transform lens for the SLM, the focal length *f* is 200 mm. The propagation distance Δ*L* is determined by the spacing between the target focal plane and the focal plane of L8 on the imaging side. Δ*L* = *M* ^2^ Δ*l*, where Δ*l* is the relative spacing to the focal plane of the objective on the object side, and *M* is the magnification of the image system. As described in Table 1, the phase patterns can be calculated based on Δ*L*. For example, for nine focal planes, if the desired focal plane spacings are *d*_1_, *d*_2_…*d*_8_, the phase pattern is determined by the following steps: (1) calculate the distance Δ*l* of each plane to the focal plane of the objective, e.g., Δ*l* = 0, *d*_1_, *d*_1_+*d*_2_, *d*_1_+*d*_2_+*d*_3_… $\sum_{i}^{8}d_{i}$, when the first plane is the focal plane of the objective; (2) calculate the propagation distance Δ*L* in the image region by Δ*L* = *M*^2^ Δ*l*; and (3) calculate the phase pattern by substituting the Δ*L* to the algorithm, i.e. step 9 in Table 1.

**Table 1:** Weighted Global Gerchberg-Saxton algorithm for MFM.

Step No.	Parameter Assignment	Remark
1		**procedure** Weighted Global GS Algorithm
2	*φ*_0_	**Initialized** by superposition
3	n←1	Iteration number assignment
4	**while** *n*<max	**Iteration do** from **1** to ***max***
5	*φ*_*n*_←*φ*_*n-*1_	
6	*E*_*A*_←*E*_0_exp(*iφ*_*n*_)	*E*_0_ is the amplitude distribution of input plane wave
7	$E_{B}\leftarrow F\left(E_{A}\right)$	
8	***for*** *i =* 1:1: ***Num of planes***	**Iteration do** from **1** to ***Num of planes***
9	$E_{C}\leftarrow T(E_{B},\Delta L\left(i\right))$	
10	*E*_*D*_←*w*(*i*)*E*_*tar*_(*i*)exp(*i*Arg(*E*_*C*_))	*w*(*i*) is the weighting factor of each focal plane; *E*_*tar*_ is the amplitude distribution of the target plane
11	$E_{E}\leftarrow T\left(E_{D,}‐\Delta L\left(i\right)\right)$	
12	$E_{F}\left(i\right)\leftarrow F^{‐1}\left(E_{E}\right)$	
13	*E*_*G*_←Σ_*i*_*E*_*F*_(*i*)	End **for**
14	*φ*_*n*_←Arg(*E*_*G*_)	End **while** when n = ***max***
15	*φ*_*n*_	**Return**

**Fig 2:**
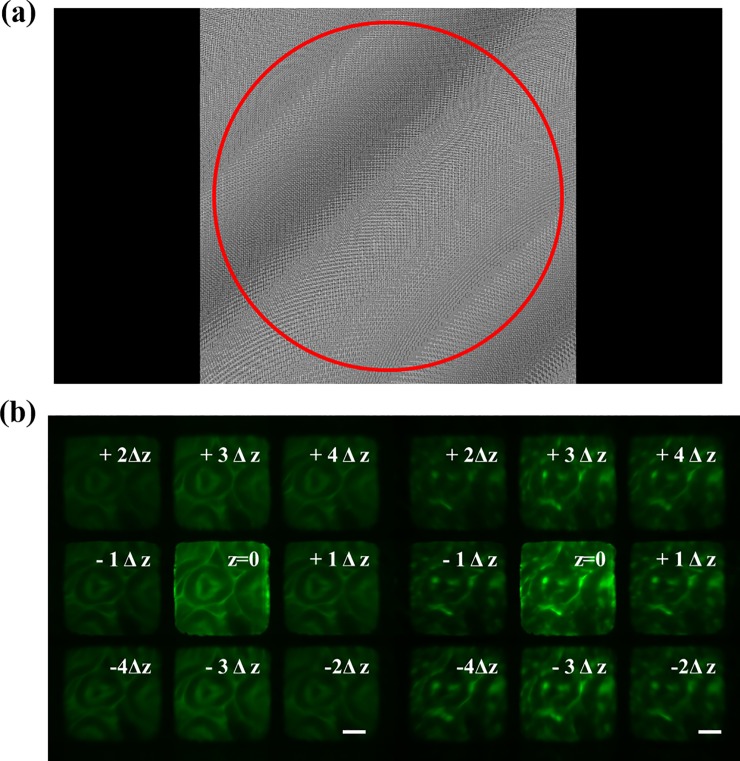
Calculated phase pattern and the raw images with uniform and speckle illumination (a) Phase pattern for the MFM calculated based on the weighted global GS algorithm, where the red circle represents the back aperture area relayed from the objective; (b) raw images of mouse kidney slices under uniform illumination (left) and speckle illumination (right). Scale bar = 15 μm.

Fig 2(A) presents a representative phase pattern for the SLM calculated based on the algorithm, where the red circle represents the aperture area relayed from the back aperture of the objective; this ensures all frequencies of the image collected by the objective can be modulated by the SLM. Fig 2(B) presents the imaging results of a mouse kidney slice (F24630, Invitrogen, USA) at nine different focal planes via the MFM under uniform (left) and speckle illumination (right) respectively, where the distance Δ*z* between each plane is set as 0.4 μm, i.e., Δ*l = -*4Δ*z*, *-*3Δ*z*…3Δ*z*, and 4Δ*z*, respectively for each focal plane. The images are acquired by the sCMOS camera with an exposure time of 30 ms, where emissions from the nine focal planes are multiplexed and imaged to different regions of the sCMOS chip. From Fig 2(B), it is observed that the structure of the mouse kidney gradually varies from -4Δ*z* to 4Δ*z*, demonstrating the depth of field multiplexing capability of the MFM. We also observe that the images from different focal planes have a uniform intensity distribution, except the central one which does not undergo the spherical phase modulation. This is because most photons collected in the center image are directly reflected by the cover glass of the SLM; the intensity of this image is higher than others even though the weight of this focal plane is set as zero when designing the phase pattern. This issue can be overcome by driving the SLM with an optimal *γ-*curve or apply anti-reflection coatings to the SLM chip cover. By fusing the two raw images collected at each focal plane via the HiLo algorithm, fast high-resolution 3-D imaging can be realized. It is worthwhile to note that the distance between each focal plane can be arbitrarily adjusted by using different phase patterns.

### HiLo algorithm for background noise rejection

HiLo algorithm is an efficient method to reject background noises in fluorescent microscopy. The algorithm is used in the MFM system as it only requires two raw images (obtained via uniform and speckle illuminations) that improves the spatial resolution without sacrificing too much temporal resolution comparing with typical three-snapshot algorithms. The main idea of the HiLo algorithm is to only modulate the in-focus image via speckle patterns to reconstruct a high-resolution image. The image *I*_*u*_ with uniform illumination acquired by the sCMOS camera can be expressed as:

$$I_{u}(\vec{r})=I_{in}(\vec{r})+I_{out}(\vec{r})$$ (3)

where $\vec{r}$ is the coordinates of the image. *I*_*in*_ and *I*_*out*_ represent the in-focus and out-of-focus emissions, respectively. The image *I*_*s*_ with speckle illumination can be expressed as:

$$I_{s}(\vec{r})=I_{in}(\vec{r})S(\vec{r})+I_{out}(\vec{r})$$ (4)

where *S* denotes the speckle modulation introduced by the speckle illumination. The difference between the two images, i.e., $I_{D}(\vec{r})=I_{s}(\vec{r})-I_{u}(\vec{r})=(S(\vec{r})-1)I_{in}(\vec{r})$, removes the out-of-focus emission term in Eqs (3) and (4). The low frequency contains the DC frequency of the optically sectioned image, i.e., *I*_*in*_. As out-of-focus noises are generally of low frequency, the high frequency part of in-focus image can be obtained by applying a high-pass filter to *I*_*u*_. Hence, a HiLo image *I*_*HiLo*_ that rejects background noises can be achieved and expressed as [22]:

$$I_{HiLo}(\vec{r})=\eta I_{lp}(\vec{r})+I_{hp}(\vec{r})$$ (5)

where *I*_*lp*_ is the low pass filtered result of *I*_*D*_; *I*_*hp*_ is the high pass filtered result of *I*_*u*_, which can be respectively expressed as $I_{lp}(\vec{r})=F^{-1}(I_{D}(\vec{\kappa })LP(\vec{\kappa }))$ and $I_{hp}(\vec{r})=F^{-1}(I_{u}(\vec{\kappa })HP(\vec{\kappa }))$; and *η* is the scaling factor. $I_{D}(\vec{\kappa })$ and $I_{u}(\vec{\kappa })$ are the frequency spectra of *I*_*D*_ and *I*_*u*_, respectively; $\vec{\kappa }$ denotes the spatial frequency. $LP(\vec{\kappa })$ and $HP(\vec{\kappa })$ are the low pass filtering and high pass filtering steps in the frequency domain. In our algorithm, $LP(\vec{\kappa })$ is a Gaussian filter and $HP(\vec{\kappa })=1-LP(\vec{\kappa })$. The coefficient $\eta =|I_{u}(\vec{\kappa })HP(\vec{\kappa })|/|I_{D}(\vec{\kappa })LP(\vec{\kappa })|$.

**Fig 3:**
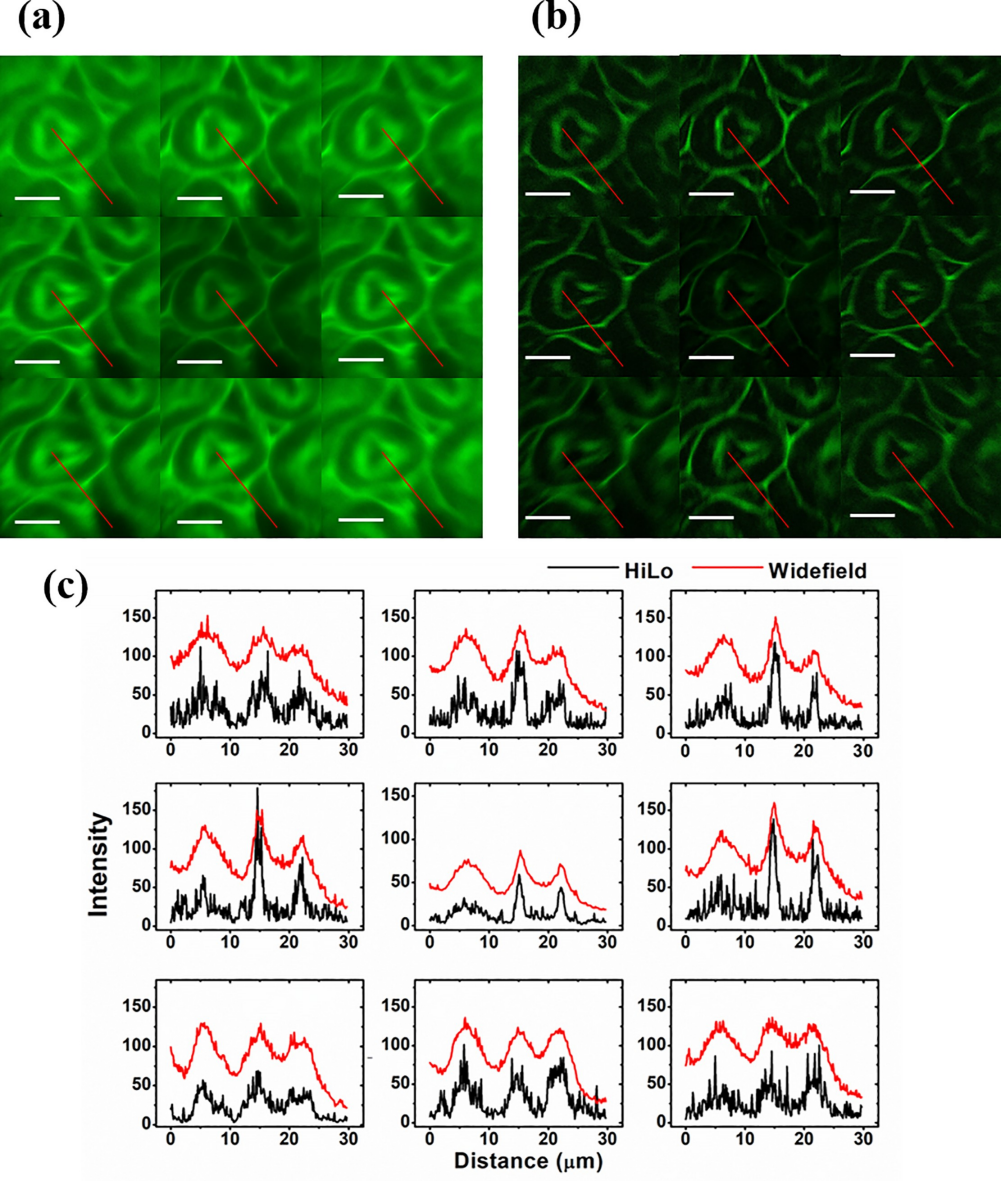
Widefield imaging and HiLo background rejection of mouse kidney via MFM (a) Widefield images at each focal plane obtained by the MFM; (b) reconstructed HiLo images that reject background emissions; (c) intensity distribution versus the corresponding red lines labeled in (a) and (b). Scale bar = 15 μm.

In this section, we reconstruct HiLo images from the nine focal planes to reject background noises using the raw images in Fig 2(B). Conventional widefield images (cropped and normalized) are provided for comparison. Fig 3(A) and 3(B) present the reconstructed widefield and HiLo images at the nine focal planes respectively. The size of each image is 54.3×54.3 μm^2^ (corresponding to 501 pixels × 501 pixels on the camera chip) with a magnification factor of 60. It can be observed in Fig 3(A) that the central image has less out-of-focus emissions compared with the other neighboring images, and the corresponding HiLo image in Fig 3(B) has better signal-to-noise ratio (SNR). To further evaluate the performance of HiLo algorithm for background noise rejection, Fig 3(C) plots the intensity distribution along the red lines at each focal plane as indicated in Fig 3(A) and 3(B). From Fig 3(C), one can clearly observe that out-of-focus emissions have been effectively removed in all HiLo images, which verifies the background noise rejection capability of the HiLo algorithm in the custom-built MFM system.

## Results and discussions

**Fig 4:**
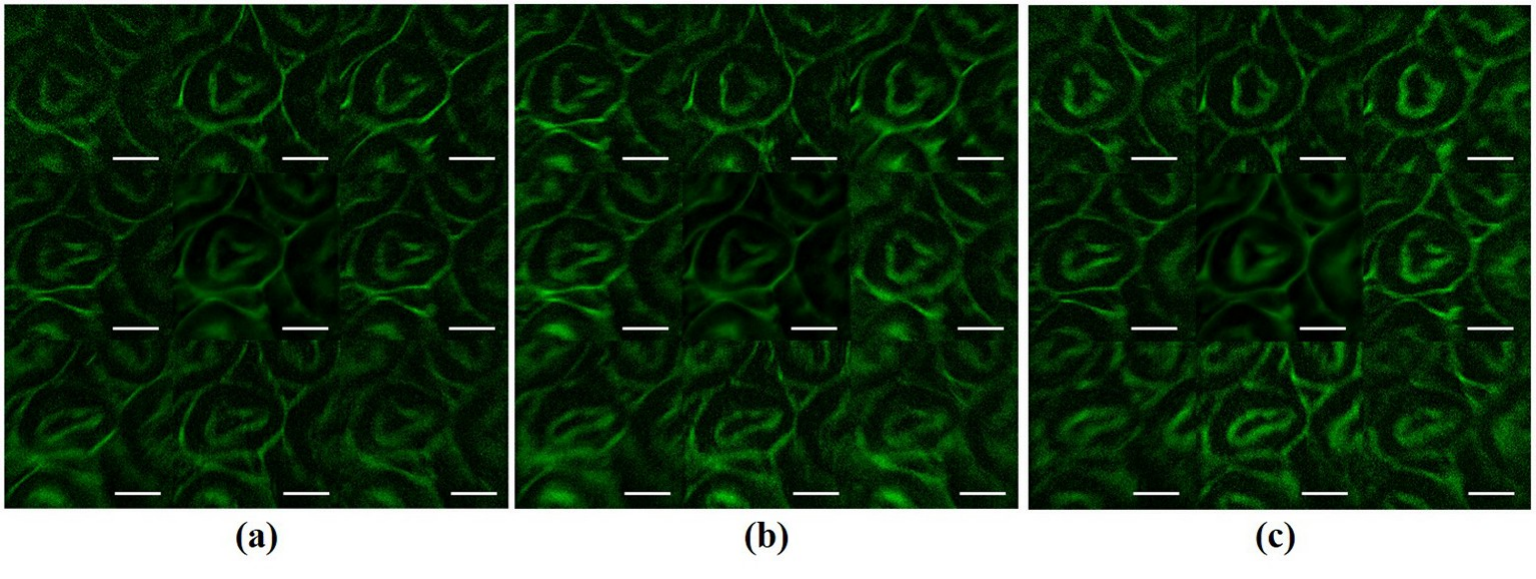
Reconstructed HiLo images from the nine focal planes with different layer spacing: (a) 0.5 μm; (b) 1.0 μm; and (c) 1.5 μm. Scale bar = 15 μm.

Comparing with grating-based MFM systems, one distinctive advantage of the new HiLo-MFM system is that the distances between the different focal planes can be arbitrarily selected and adjusted by applying different phase patterns to the SLM. To demonstrate this capability, we record raw images with the following different focal plane distances: 0.5, 1.0, and 1.5 μm. Fig 4 presents the reconstructed HiLo images. In all experiments, the exposure time for each raw image is 30 ms. (Each raw image has a size of 54.3×54.3 μm^2^.) From Fig 4, one may observe that the structures in the specimen gradually vary as the imaging depth varies. By comparing Fig 4(A), 4(B) and 4(C), one may also find the differences between the adjacent focal planes become more and more prominent when the focal plane spacing increases. As the axial position of different focal planes can be individually and arbitrarily controlled, one may use the HiLo MFM system to study fast biological events at different depths simultaneously, i.e., the different planes are distant to each other. One limitation of the method is that when the focal planes displace away from the zero position (i.e., non-modulation focal plane), the aberration introduced by the objective lens may become increasingly prominent. This issue can be addressed by adding a wavefront correction phase to the modulation of each focal plane when calculating the SLM patterns. [28]

**Fig 5:**
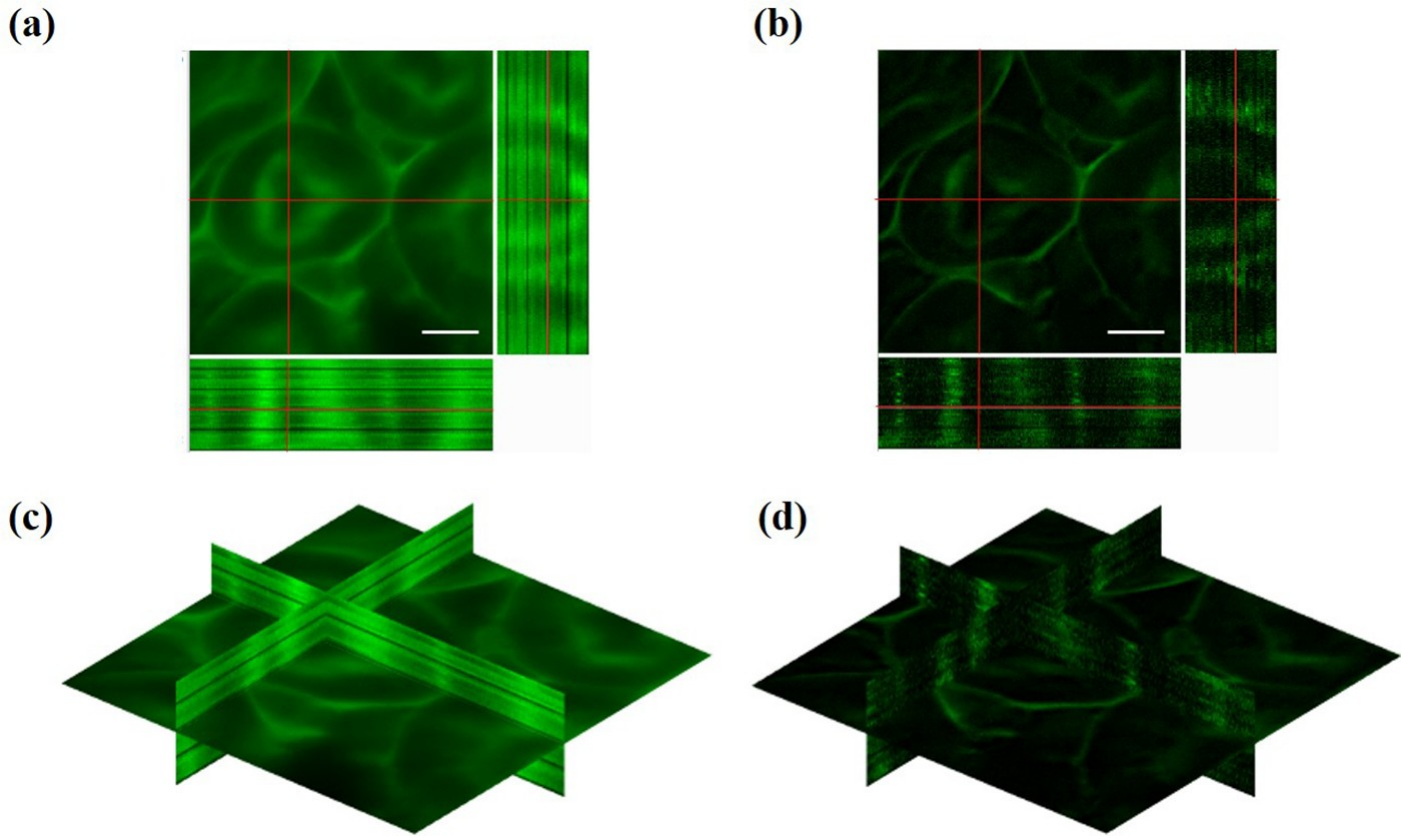
Reconstructed Cross-sectional views of the mouse kidney sample. Cross-sectional views in the x-y, x-z and y-z planes based on (a) uniform illumination and (b) HiLo algorithm; isometric cross-sectional views based on (c) uniform illumination and (d) HiLo algorithm. Scale bar = 15 μm.

**Fig 6:**
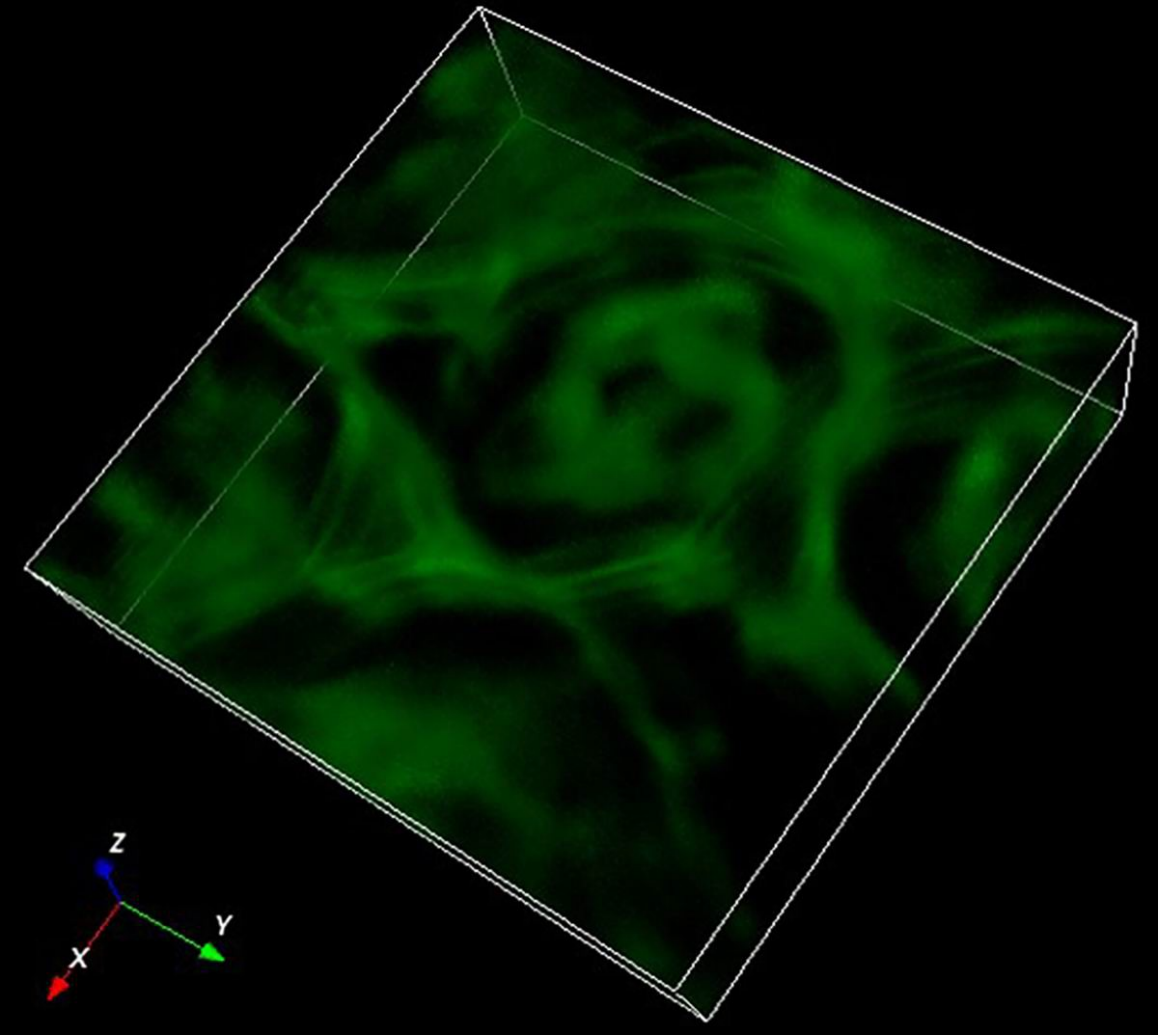
An isometric view of the constructed mouse kidney specimen based on the HiLo algorithm.

Next, we demonstrate 3-D imaging over a larger volume, i.e., 16 μm-thick mouse kidney sample, by axially scanning and stacking up the nine focal planes. The axial scanning is performed via the xyz stage with a resolution of 40 nm in the axial direction. For every two exposures (i.e., uniform and speckle illuminations), a HiLo image volume of 54.3×54.3×9Δz can be collected and reconstructed, where Δz is the distance between each focal plane. As the stage has a minimum step size of 40 nm, Δz is selected to be 0.4 μm so that the required scanning distance for each volume capture will be multiples of positive integers, i.e., 0.4 μm×9 layers×n, *n* = positive integer. To image over a depth of 16 μm, the nine focal planes (i.e., 54.3×54.3×3.6 μm^3^) will be scanned axially for five times, requiring a total of 10 raw images and generating a volume imaging of 45 sections. (Note that the last five images are disposed as they image outside the specimen.) Lastly, the collected raw images are processed by the HiLo algorithm. By stacking up the collected 40 optical sections, a 3-D image of the kidney specimen can be formed; the total pixel size of the 3-D image is 501×501×40, or equivalently 54.3×54.3×16.0 μm^3^. The results are presented in Fig 5. To better assess the background noise rejection capability of the HiLo-MFM system, we compare the reconstructed 3-D image with uniform illumination, i.e., Fig 5(A) and 5(C), with the HiLo reconstructed images, i.e., Fig 5(B) and 5(D). From the comparison, we can confirm that the background emissions have been effectively removed, and the contrast of the optical cross-sections have been substantially enhanced in all directions, i.e., in the x-y plane, x-z plane, and y-z plane. An isometric view of the reconstructed mouse kidney specimen is presented in Fig 6, where the 3-D rendering is performed via ImageJ (open source program for image processing, NIH). (See S1 Video for a 3-D rotational view of the reconstructed sample.) The imaging results in Figs 5 and 6 show that fine structures of the mouse kidney can be clearly observed with background noises suppressed by the HiLo algorithm, which well demonstrates the resolution and noise rejection capability of the HiLo-MFM system. Note that the image stacking process may introduce artifacts in the axial direction; also, the flatness of the image in the axial direction can be improved by integral normalization and 3-D interpolation processes.

The imaging speed and resolution of the HiLo-MFM system are determined by (1) the number of focal planes, which can be flexibly adjusted by using different SLM patterns and dividing the imaging chip into different segments (e.g., 2×2 or 4×4); and (2) the speed of the camera. If nine focal planes are used and the camera is recording at 200 Hz; the volume imaging rate will be 100 Hz over a volume of ~ 54.3×54.3×9.0 μm^3^ (assuming Δz = 1 μm.), corresponding to a pixel resolution of 501×501×9 pixels. Increasing the number of focal planes will increase the imaging speed at the expense of (1) reduced pixel resolution as the total pixels on a CCD chip is constant; and (2) the efficiency of each focal plane, which can be relieved by choosing detectors of higher quantum efficiency. To work with high imaging speed, the mechanical scanner in our system may be replaced by an electrically tunable lens (ETL) [10] or a tunable acoustic gradient index of refraction (TAG) lens [29]. In summary, the HiLo-MFM system presents a simple and effective solution to enable (1) trade-offs between imaging speed and resolution, and (2) simultaneous real-time imaging at selected focal plane at different depths, which may find useful bio-imaging applications.

Like other MFM systems, the resolution of the HiLo-MFM system is also influenced by the chromatic aberration issue. To address this matter, a BPF is included in our optical system to reduce chromatic aberrations, as indicated in Fig 1. Several methods may further reduce the chromatic aberration including (1) the application of BPFs with a narrower bandwidth, which simultaneously reduces the image SNR due to the reduced emissions collected in one exposure. Nevertheless, our HiLo-MFM with a narrower bandwidth BPF is suitable for analyzing the fluorescence enhanced bio-sample, such as cellular laser [30]; and (2) the application of a compensation grating. This mothed can fully address the chromatic aberration problem [11]. However, the grating needs to be custom-built and only works for a specific configuration, i.e., a new grating is needed if the number and spacing of focal planes change, making the system inflexible. In addition, deblurring algorithms may be applied in post image processing steps to further improve the image quality [31].

Efficiency has always been an important issue for different multi-layer imaging systems. In our setup, the system efficiency is estimated to be ~20% at 550 nm, which is limited by the SLM, BPF and the polarizer. The efficiency may be improved by using an SLM with higher efficiency (> 90%) and BPF with higher transmittance. Note that the efficiency of our SLM is 67%. This is achieved by setting the zero order beam as one of the focal planes; the high order diffractions are minimized by using the designed patterns based on the weighted global GS algorithm.

## Conclusions

We have presented a fast 3-D fluorescent imaging system, i.e., HiLo-MFM, based on multi-focus imaging and HiLo algorithm for background noise rejection, which presents an effective way to trade-off the imaging speed and resolution and to study fast biological events at different depths simultaneously. Multi-focus imaging is achieved by applying different phase patterns to an SLM, allowing arbitrary control of the number and positions of focal planes. The HiLo algorithm has been applied in the MFM system to reject the background emissions at each focal plane. Comparing with uniform illumination MFM, the HiLo-MFM effectively removes the background noises with substantially enhanced contrast at all focal planes. 3-D imaging experiments have been performed on a mouse kidney sample to demonstrate the enhanced resolution and contrast as well as the imaging speed, which is improved by a factor of 4.5. The new HiLo-MFM system may find important bio-imaging applications that require high imaging speed and simultaneous investigation at different depths, e.g., imaging neural circuits in a mouse brain.

## Supporting information

S1 Video 3-D rotational view of the reconstructed sample. (MP4)
